# Professor M‘Clintock’s Introductory Lecture

**Published:** 1842-05-14

**Authors:** 


### THE MEDICAL EXAMINER.

### PHILADELPHIA, MAY 14, 1842.

*Professor M'Clintock's Introductory Lecture.*—This lecture was delivered at the opening of the session at the Castleton Medical College, Vermont, on the 8th of March, by Professor JAMES M'CLINTOCK, formerly of Philadelphia. It is an appropriate and well digested introductory to a course of medicine, written in a plain, energetic style, free from flourish, practical, and to the point. The professor discusses the relative advantages of metropolitan and village instruction to the student of medicine, and, though stating the case in the main very fairly, strikes the balance in favour of the latter. He pays a handsome compliment to "Philadelphia, the metropolis of Medical Science in this country, the only city in which medical schools have ever obtained the highest rank, or kept up a healthy existence for any length of time," and does ample justice to the opportunities for instruction that are here afforded. On the side of the village schools, Castleton in particular, he offers cheapness, freedom from the allurements of dissipation, and a course (we have no doubt a good one) of *eight* lectures. "In facilities for clinical instruction, he does not pretend to compare with Philadelphia," and herein, indeed, lies the great and hopeless inferiority of very small towns, as sources of practical instruction. Clinical medicine, and, especially, pathological anatomy, can hardly be taught except in large hospitals, and every student of medicine should endeavour to place these means of instruction within his reach, at least during a portion of his pupilage. The ground-work of a good medical education may no doubt be laid at schools like Castleton—we know at least that Prof. M'Clintock is an excellent teacher of anatomy; but he must excuse us for insisting that Philadelphia and some other large towns offer more advantages for *finishing off* pupils.

